# Supplementary material for: A methodological and reporting quality assessment of systematic reviews/meta-analyses on exercise interventions for cognitive function in older adults with mild cognitive impairment
Source: PeerJ. 2024 Jul 24;12:e17773. doi: 10.7717/peerj.17773 (PMC11283171; doi:10.7717/peerj.17773)
Supplement: Supplemental Information 1 [file peerj-12-17773-s001.docx]

**Delete references after reading the full text**

| **1** | **Karssemeijer EGA, Aaronson JA, Bossers WJ, Smits T, Olde Rikkert MGM, Kessels RPC. Positive effects of combined cognitive and physical exercise training on cognitive function in older adults with mild cognitive impairment or dementia: A meta-analysis. Ageing Res Rev. 2017 Nov;40:75-83.** | **Subjects did not meet inclusion criteria.** |
| --- | --- | --- |
| **2** | **Blackman J, Swirski M, Clynes J, Harding S, Leng Y, Coulthard E. Pharmacological and non-pharmacological interventions to enhance sleep in mild cognitive impairment and mild Alzheimer's disease: A systematic review. J Sleep Res. 2021 Aug;30(4):e13229.** | **Subjects did not meet inclusion criteria.** |
| **3** | **Gavelin HM, Dong C, Minkov R, Bahar-Fuchs A, Ellis KA, Lautenschlager NT, Mellow ML, Wade AT, Smith AE, Finke C, Krohn S, Lampit A. Combined physical and cognitive training for older adults with and without cognitive impairment: A systematic review and network meta-analysis of randomized controlled trials. Ageing Res Rev. 2021 Mar;66:101232.** | **Subjects did not meet inclusion criteria.** |
| **4** | **Ali N, Tian H, Thabane L, Ma J, Wu H, Zhong Q, Gao Y, Sun C, Zhu Y, Wang T. The Effects of Dual-Task Training on Cognitive and Physical Functions in Older Adults with Cognitive Impairment; A Systematic Review and Meta-Analysis. J Prev Alzheimers Dis. 2022;9(2):359-370.** | **Subjects did not meet inclusion criteria.** |
| **5** | **Wayne PM, Walsh JN, Taylor-Piliae RE, Wells RE, Papp KV, Donovan NJ, Yeh GY. Effect of tai chi on cognitive performance in older adults: systematic review and meta-analysis. J Am Geriatr Soc. 2014 Jan;62(1):25-39.** | **Subjects did not meet inclusion criteria.** |
| **6** | **Lam FM, Huang MZ, Liao LR, Chung RC, Kwok TC, Pang MY. Physical exercise improves strength, balance, mobility, and endurance in people with cognitive impairment and dementia: a systematic review. J Physiother. 2018 Jan;64(1):4-15.** | **Outcome indicators did not meet the inclusion criteria.** |
| **7** | **Li H, Su W, Dang H, Han K, Lu H, Yue S, Zhang H. Exercise Training for Mild Cognitive Impairment Adults Older Than 60: A Systematic Review and Meta-Analysis. J Alzheimers Dis. 2022;88(4):1263-1278.** | **Outcome indicators did not meet the inclusion criteria.** |
| **8** | **Yan J, Li X, Guo X, Lin Y, Wang S, Cao Y, Lin H, Dai Y, Ding Y, Liu W. Effect of Multicomponent Exercise on Cognition, Physical Function and Activities of Daily Life in Older Adults With Dementia or Mild Cognitive Impairment: A Systematic Review and Meta-analysis. Arch Phys Med Rehabil. 2023 Dec;104(12):2092-2108.** | **Subjects did not meet inclusion criteria.** |
| **9** | **Ma C, Lin M, Gao J, Xu S, Huang L, Zhu J, Huang J, Tao J, Chen L. The impact of physical activity on blood inflammatory cytokines and neuroprotective factors in individuals with mild cognitive impairment: a systematic review and meta-analysis of randomized-controlled trials. Aging Clin Exp Res. 2022 Jul;34(7):1471-1484.** | **Outcome indicators did not meet the inclusion criteria.** |
| **10** | **Wang YY, Wang XX, Chen L, Liu Y, Li YR. A systematic review and network meta-analysis comparing various non-pharmacological treatments for older people with mild cognitive impairment. Asian J Psychiatr. 2023 Aug;86:103635.** | **Interventions did not meet inclusion criteria.** |
| **11** | **Abd-Alrazaq A, Abuelezz I, AlSaad R, Al-Jafar E, Ahmed A, Aziz S, Nashwan A, Sheikh J. Serious Games for Learning Among Older Adults With Cognitive Impairment: Systematic Review and Meta-analysis. J Med Internet Res. 2023 Apr 12;25:e43607.** | **Interventions did not meet inclusion criteria.** |
| **12** | **Lin M, Ma C, Zhu J, Gao J, Huang L, Huang J, Liu Z, Tao J, Chen L. Effects of exercise interventions on executive function in old adults with mild cognitive impairment: A systematic review and meta-analysis of randomized controlled trials. Ageing Res Rev. 2022 Dec;82:101776.** | **Outcome indicators did not meet the inclusion criteria.** |
| **13** | **Gavelin HM, Dong C, Minkov R, Bahar-Fuchs A, Ellis KA, Lautenschlager NT, Mellow ML, Wade AT, Smith AE, Finke C, Krohn S, Lampit A. Combined physical and cognitive training for older adults with and without cognitive impairment: A systematic review and network meta-analysis of randomized controlled trials. Ageing Res Rev. 2021 Mar;66:101232.** | **Subjects did not meet inclusion criteria.** |
| **14** | **Wang YQ, Jia RX, Liang JH, Li J, Qian S, Li JY, Xu Y. Effects of non-pharmacological therapies for people with mild cognitive impairment. A Bayesian network meta-analysis. Int J Geriatr Psychiatry. 2020 Jun;35(6):591-600.** | **Subjects did not meet inclusion criteria.** |
| **15** | **Liu Q, Ni W, Zhang L, Zhao M, Bai X, Zhang S, Ding Y, Yin H, Chen L. Comparative efficacy of various exercise interventions on depression in older adults with mild cognitive impairment: A systematic review and network meta-analysis. Ageing Res Rev. 2023 Nov;91:102071.** | **Outcome indicators did not meet the inclusion criteria.** |
| **16** | **Liu H, Song Y, Zhao D, Zhan M. Effect of exercise on cognitive impairment in patients undergoing haemodialyses: A systematic review and meta-analysis of randomised controlled trials. J Ren Care. 2022 Dec;48(4):243-252.** | **Subjects did not meet inclusion criteria.** |
| **17** | **Hu M, Hu H, Shao Z, Gao Y, Zeng X, Shu X, Huang J, Shen S, Wu IXY, Xiao LD, Feng H. Effectiveness and acceptability of non-pharmacological interventions in people with mild cognitive impairment: Overview of systematic reviews and network meta-analysis. J Affect Disord. 2022 Aug 15;311:383-390.** | **Outcome indicators did not meet the inclusion criteria.** |
| **18** | **Dieckelmann M, González-González AI, Banzer W, Berghold A, Jeitler K, Pantel J, Pregartner G, Schall A, Tesky VA, Siebenhofer A. Effectiveness of exercise interventions to improve long-term outcomes in people living with mild cognitive impairment: a systematic review and meta-analysis. Sci Rep. 2023 Oct 23;13(1):18074.** | **Outcome indicators did not meet the inclusion criteria.** |
| **19** | **Dequanter S, Gagnon MP, Ndiaye MA, Gorus E, Fobelets M, Giguère A, Bourbonnais A, Buyl R. The Effectiveness of e-Health Solutions for Aging With Cognitive Impairment: A Systematic Review. Gerontologist. 2021 Sep 13;61(7):e373-e394.** | **Interventions did not meet inclusion criteria.** |
| **20** | **Di Lorito C, Bosco A, Booth V, Goldberg S, Harwood RH, Van der Wardt V. Adherence to exercise interventions in older people with mild cognitive impairment and dementia: A systematic review and meta-analysis. Prev Med Rep. 2020 Jun 1;19:101139.** | **Subjects did not meet inclusion criteria.** |
| **21** | **Di Lorito C, Bosco A, Booth V, Goldberg S, Harwood RH, Van der Wardt V. Adherence to exercise interventions in older people with mild cognitive impairment and dementia: A systematic review and meta-analysis. Prev Med Rep. 2020 Jun 1;19:101139.** | **Subjects did not meet inclusion criteria.** |
| **22** | **Shao Z, Hu M, Zhang D, Zeng X, Shu X, Wu X, Kwok TCY, Feng H. Dose-response relationship in non-pharmacological interventions for individuals with mild cognitive impairment: A systematic review and meta-analysis of randomised controlled trials. J Clin Nurs. 2022 Dec;31(23-24):3390-3401.** | **Outcome indicators did not meet the inclusion criteria.** |
| **23** | **Meng Q, Yin H, Wang S, Shang B, Meng X, Yan M, Li G, Chu J, Chen L. The effect of combined cognitive intervention and physical exercise on cognitive function in older adults with mild cognitive impairment: a meta-analysis of randomized controlled trials. Aging Clin Exp Res. 2022 Feb;34(2):261-276.** | **Interventions did not meet inclusion criteria.** |
| **24** | **Zhao Y, Feng H, Wu X, Du Y, Yang X, Hu M, Ning H, Liao L, Chen H, Zhao Y. Effectiveness of Exergaming in Improving Cognitive and Physical Function in People With Mild Cognitive Impairment or Dementia: Systematic Review. JMIR Serious Games. 2020 Jun 30;8(2):e16841.** | **Subjects did not meet inclusion criteria.** |
| **25** | **Jiang J, Guo W, Wang B. Effects of exergaming on executive function of older adults: a systematic review and meta-analysis. PeerJ. 2022 Apr 11;10:e13194.** | **Interventions did not meet inclusion criteria.** |
| **26** | **Öhman H, Savikko N, Strandberg TE, Pitkälä KH. Effect of physical exercise on cognitive performance in older adults with mild cognitive impairment or dementia: a systematic review. Dement Geriatr Cogn Disord. 2014;38(5-6):347-65.** | **Interventions did not meet inclusion criteria.** |
| **27** | **The influence of exercise interventions on cognitive functions in patients with amnestic mild cognitive impairment: A systematic review and meta-analysis** | **Subjects did not meet inclusion criteria.** |
| **28** | **Deng J, Wang H, Fu T, Xu C, Zhu Q, Guo L, Zhu Y. Physical activity improves the visual-spatial working memory of individuals with mild cognitive impairment or Alzheimer's disease: a systematic review and network meta-analysis. Front Public Health. 2024 Mar 28;12:1365589.** | **Interventions did not meet inclusion criteria.** |
| **29** | **Ströhle A, Schmidt DK, Schultz F, Fricke N, Staden T, Hellweg R, Priller J, Rapp MA, Rieckmann N. Drug and Exercise Treatment of Alzheimer Disease and Mild Cognitive Impairment: A Systematic Review and Meta-Analysis of Effects on Cognition in Randomized Controlled Trials. Am J Geriatr Psychiatry. 2015 Dec;23(12):1234-1249.** | **Subjects did not meet inclusion criteria.** |
| **30** | **Yang C, Moore A, Mpofu E, Dorstyn D, Li Q, Yin C. Effectiveness of Combined Cognitive and Physical Interventions to Enhance Functioning in Older Adults With Mild Cognitive Impairment: A Systematic Review of Randomized Controlled Trials. Gerontologist. 2020 Nov 23;60(8):633-642.** | **Subjects did not meet inclusion criteria.** |
